# Global remote sensing of water–chlorophyll ratio in terrestrial plant leaves

**DOI:** 10.1002/ece3.361

**Published:** 2012-09-13

**Authors:** Keiji Kushida

**Affiliations:** Center for Far Eastern Studies and Graduate School of Science and Engineering (Science), University of Toyama3190 Gofuku, Toyama, 930-8555, Japan

**Keywords:** Leaf area index, leaf chlorophyll concentration, leaf water concentration, radiative transfer, remote sensing

## Abstract

I evaluated the use of global remote sensing techniques for estimating plant leaf chlorophyll *a* + *b* (*C*_ab_; μg cm^−2^) and water (*C*_w_; mg cm^−2^) concentrations as well as the ratio of *C*_w_/*C*_ab_ with the PROSAIL model under possible distributions for leaf and soil spectra, leaf area index (LAI), canopy geometric structure, and leaf size. First, I estimated LAI from the normalized difference vegetation index. I found that, at LAI values <2, *C*_ab_, *C*_w_, and *C*_w_/*C*_ab_ could not be reliably estimated. At LAI values >2, *C*_ab_ and *C*_w_ could be estimated for only restricted ranges of the canopy structure; however, the ratio of *C*_w_/*C*_ab_ could be reliably estimated for a variety of possible canopy structures with coefficients of determination (*R*^2^) ranging from 0.56 to 0.90. The remote estimation of the *C*_w_/*C*_ab_ ratio from satellites offers information on plant condition at a global scale.

## Introduction

Leaf chlorophyll *a* + *b* (*C*_ab_; μg cm^−2^), dry matter (*C*_m_; mg cm^−2^), and water (*C*_w_; mg cm^−2^) concentrations provide information on plant physiological status and ecosystem functioning (e.g., terrestrial heat, water, and CO_2_ balances) (Zarco-Tejada et al. 2003). Nevertheless, global remote estimates of vegetation status mainly focus on leaf area index (LAI; Garrigues et al. 2008), fraction of absorbed photosynthetically active radiation (FAPAR; Running et al. 2004), phenology (Zhang et al. 2006), leaf clumping (Chen et al. 2005), and vegetation height (Simard et al. 2011).

Site-specific studies have clarified the relationships between plant canopy spectral reflectance and *C*_ab_ (Zarco-Tejada et al. 2004; Gitelson et al. 2005; Darvishzadeh et al. 2012; Si et al. 2012), *C*_m_, (Fourty and Baret 1997) and *C*_w_ (Bowyer and Danson 2004; De Santis et al. 2006; Zarco-Tejada et al. 2003). However, global or regional relationships between these factors are not clearly understood because other characteristics of the plant canopy impact those relationships. In this note, I evaluated the use of global remote sensing techniques for estimating *C*_ab_, *C*_m_, *C*_w_, and the *C*_w_/*C*_ab_ ratio using the PROSAIL model (Jacquemoud et al. 2009) at varying possible distributions of leaf and soil spectra, LAI, canopy geometric structure, and leaf size.

## Materials and Methods

I used the PROSAIL model, which is a combination of the SAIL (Verhoef 1984) and PROSPECT (Jacquemoud and Baret 1990) models, for calculating the relationships between the top-of-the-atmosphere (TOA) canopy spectral reflectance and *C*_ab_, *C*_m_, *C*_w_, and *C*_w_/*C*_ab_. The input parameters of the PROSAIL model are listed in Table 1, and I noted TOA canopy spectral reflectance from the model outputs.

**Table 1:** Input parameters for the PROSAIL model

	Unit	Value/function
ln(*C*_ab_)	μg cm^−2^	*N*(3.79, 0.35^2^)*
ln(*C*_m_)	mg cm^−2^	*N*(1.57, 0.42^2^)*
ln(*C*_w_)	mg cm^−2^	*N*(2.32, 0.47^2^)*
The coefficient of correlation between ln(*C*_ab_) and ln(*C*_m_)	–	0.56
The coefficient of correlation between ln(*C*_w_) and ln(*C*_ab_) or ln(*C*_m_)	–	0
The parameter characterizing the leaf mesophyll structure, *N*	–	*N*(1.7, 0.2^2^)*
Soil reflectance	–	JHU-SL based statistic model
Clumping index, *Ω*	–	Any (fixed at 0.7 for LAI calculation)
Leaf angle distribution, LAD	–	Erectophile, spherical, plagiophile, uniform, extremophile, and planophile
Canopy hotspot parameter, *S*_l_	–	0.0001, 0.001, 0.01, and 0.1
Leaf area index, *LAI* = *LAI*_*r*_ • *Ω*	–	0–10 (10^−6^ interval)
Solar incident zenith angle, *θ*_s_	°	25 and 50
View zenith angle, *θ*_v_	°	0
Solar illumination specular ratio, *r*_sd_	–	Constant (0.81, 0.91, 0.95, 0.98, and 1.0 in MODIS bands 3, 4, 1, 2, and 5–7)
Error function of the atmospheric correction	–	*N*(0, (0.005 + 0.05*ρ*)^2^)*

* *N*(*μ*,*σ*^2^) denotes the normal distribution with mean *μ* and variance *σ*^2^.

I calculated the relationship between the normalized difference vegetation index (NDVI) and LAI (m^2^ m^−2^) following Kushida and Yoshino (2010). I used the TOA canopy spectral reflectance values at the Moderate Resolution Imaging Spectroradiometer (MODIS) red (620–670 nm; *R*_R_(%)) and near infrared (841–876 nm; *R*_NIR_(%)) bands to calculate NDVI as follows:


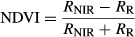
(1)

For the estimated LAI value ranges of 0.95–1.05, 1.95–2.05, 2.95–3.05, 3.95–4.05, 4.95–5.05, 5.95–6.05, and 6.95–7.05, I calculated the relationships between canopy spectral reflectance and *C*_ab_, *C*_m_, *C*_w_, and *C*_w_/*C*_ab_.

I assumed that *C*_ab_, *C*_m_, and *C*_w_ had lognormal distributions based on the leaf optical properties experiment (LOPEX93), which provides values for leaf pigment and water content of 70 leaf samples that represent approximately 50 species of woody and herbaceous plants (Hosgood et al. 1995). The means and standard deviations (SD) of the lognormal distributions and the correlations and ranges of the variables were also determined using the data values in LOPEX93. All calculations were carried out under variable soil spectrum conditions. The soil reflectance distribution for each of the bands was assumed to be lognormal or normal, based on the Johns Hopkins University Spectral Library (JHU-SL; Baldridge et al. 2009). The means and SD of the distributions and the correlations and ranges of the variables were determined using the data values in JHU-SL.

The clumping index (Ω; Chen et al. 2005) was incorporated in the model by setting the parameter at 0.7 to express the leaf clumping effect. Using this parameter, I adjusted the LAI value such that, in a plant canopy with an initial LAI value of *LAI*_r_ and an Ω value of *Ω*, the adjusted LAI value became *LAI*_r_ *· Ω*/0.7. The leaf angle distribution (LAD) was fixed as erectophile, spherical, plagiophile, uniform, extremophile, and planophile. The canopy hotspot parameter (*S*_l_), which is equal to the ratio of the correlation length of leaf projections in the horizontal plane and the canopy height, was fixed at 0.0001, 0.001, 0.01, and 0.1. I used these ranges of LAD and *S*_l_ values to represent global distributions in these parameters. The solar incident zenith angle (*θ*_s_) was fixed at 25° for all model evaluations except when model sensitivity to this parameter was evaluated, and, in this case, *θ*_s_ was increased to 50°. The specular ratio to the total solar illumination (*r*_sd_) was set at constant values representing typical atmospheric conditions on a clear day for each of the bands. I assumed that the error function of the atmospheric correction had an independent normal distribution with a mean of 0 and SD of 0.005 + 0.05*ρ* (no unit reflectance), where *ρ* is the reflectance value at a given spectral band (Vermote and Kotchenova 2008).

In the calculation of each of combinations of the LAD and *S*_l_ types, LAI values were provided from 0 to 10 at 10^−6^ intervals. I used pseudo-random numbers to express the lognormal and normal distributions of the leaf spectral parameters and soil reflectance. I calculated 10^7^ cases to obtain the relationships between *C*_ab_, *C*_m_, and *C*_w_ and their associated spectral reflectances.

I used the TOA canopy spectral reflectance values at the MODIS green (545–565 nm; *R*_G_(%)), near infrared (841–876 nm; *R*_NIR_(%)), and shortwave infrared (1628–1652 nm; *R*_1640_(%)) bands to estimate *C*_ab_, *C*_m_, and *C*_w_, respectively. The *R*_G_, *R*_NIR_, and *R*_1640_ bands correspond to absorption bands of chlorophyll *a* + *b*, dry matter, and water and dry matter combined, respectively. Absorptions of carotenoids and anthocyanins also concern with *R*_G_; however, in general, most of the leaf absorption at the green band corresponds to chlorophyll *a* + *b*. That was because the carotenoids concentration and *C*_ab_ have a high positive correlation for a variety of plant species and the anthocyanins concentration appears when the leaves of deciduous trees turned red in autumn. For the estimation of *C*_w_, the estimated *C*_m_ value from *R*_NIR_ was multiplied by the ratio of the specific absorption coefficient of water (cm^2^ mg) to that of dry matter (cm^2^ mg), which is 0.787, and then removed this value (*f*_i_(*R*_NIR_)) from the estimated *C*_w_ because the PROSPECT model shows that both *C*_w_ and *C*_m_ contribute to *R*_1640_. For the estimation of *C*_w_/*C*_ab_, the estimated *C*_w_ value was divided by the estimated *C*_ab_ value.

I divided the ranges of the values of the TOA spectral reflectance or the abovementioned spectral indices into 8–12, and then calculated the average and SD of *C*_ab_, *C*_m_, *C*_w_, and *C*_w_/*C*_ab_ for each of the divided ranges. To express the relationships in mathematical formulas for each of the estimated LAI value ranges, I used regression equations in the form:


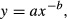
(2)

where *a* and *b* are constant values, *x* is the spectral reflectance (*R*_G_, *R*_NIR_, or *R*_1640_ *+ f*_i_(*R*_NIR_)), and *y* is the leaf constituent (*C*_ab_, *C*_m_, or *C*_w_). For the ratio *C*_w_/*C*_ab_ for each of the estimated LAI value ranges, I used the regression equation in the form:


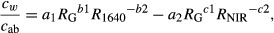
(3)

where *a*_1_, *a*_2_, *b*_1_, *b*_2_, *c*_1_, and *c*_2_ were constant values. The coefficient of determination (*R*^2^) was defined as:


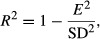
(4)

where *E* is the root mean square error (RMSE) from the regression and SD is the standard deviation of the samples. Standard deviation for *C*_ab_, *C*_m_, *C*_w_, and *C*_w_/*C*_ab_ was 16.4 μg cm^−2^, 2.22 mg cm^−2^, 5.63 mg cm^−2^, and 0.174, respectively.

## Results

**Figure 1:**
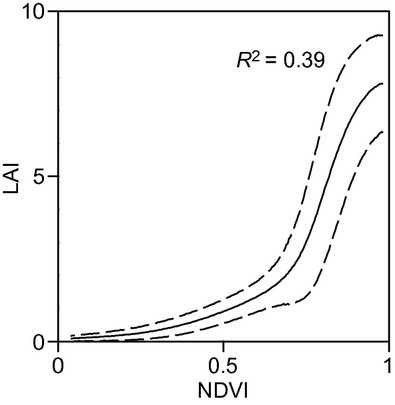
Relationship between normalized difference vegetation index and estimated leaf area index for all combinations of leaf angle distribution and *S*_l_ types (*θ*_s_ = 25°). The solid and dotted curves denote the average and the average ±SD, respectively, for each estimate.

The LAI was estimated from the NDVI with *R*^2^ of 0.39 for all combinations of the LAD and *S*_l_ types together when *θ*_s_ = 25° (Fig. 1). The SD of the estimated LAI values over the intervals 0.95–1.05, 1.95–2.05, 2.95–3.05, 3.95–4.05, 4.95–5.05, 5.95–6.05, and 6.95–7.05 were 0.4, 0.9, 1.6, 2.1, 2.2, 2.1, and 1.8, respectively. Similarly, the LAI was estimated form the NDVI with *R*^2^ of 0.30 when *θ*_s_ = 50°.

**Figure 2:**
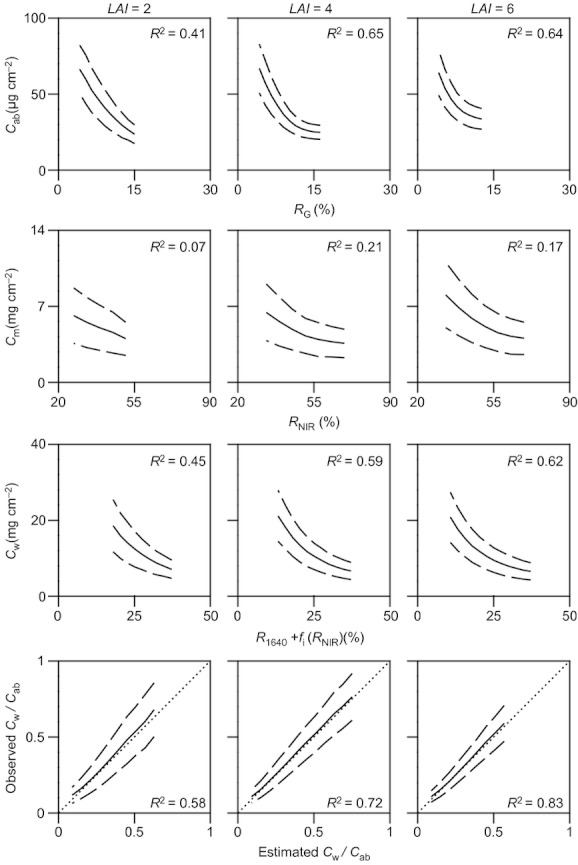
*R*_G_ versus *C*_ab_, *R*_NIR_ versus *C*_m_, *R*_1640_ + *f*_*i*_(*R*_NIR_) versus *C*_w_, and estimated *C*_w_/*C*_ab_ versus observed *C*_w_/*C*_ab_ for all combinations of leaf angle distribution and *S*_l_ types and for estimated leaf area indices of 2, 4, and 6 (*θ*_s_ = 25°). The solid and dotted curves denote the average and the average ±SD, respectively, for each estimate.

I found a weak relationship between *C*_m_ and *R*_NIR_ and a strong relationship between *C*_ab_, *C*_w_, and *C*_w_/*C*_ab_ and their associated canopy spectral reflectances at the LAI values >2 when all the LAD and *S*_l_ types were equally probable in one pixel of a remotely sensed image (Fig. 2). At the LAI values of 1, the *R*^2^ of *C*_ab_, *C*_w_, and *C*_w_/*C*_ab_ estimations were <0.22. The LAI estimates of 2, 4, and 6 in Figure 2 correspond to the estimated LAI ranges of 1.95–2.05, 3.95–4.05, and 5.95–6.05, respectively.

**Figure 3:**
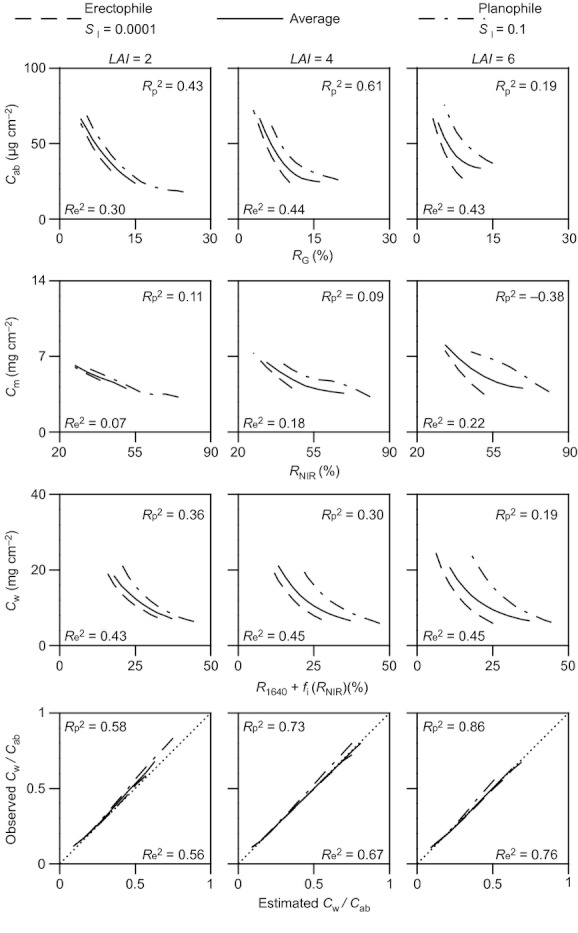
*R*_G_ versus *C*_ab_, *R*_NIR_ versus *C*_m_, *R*_1640_ + *f*_*i*_(*R*_NIR_) versus *C*_w_, and estimated *C*_w_/*C*_ab_ versus observed *C*_w_/*C*_ab_ for estimated leaf area indices of 2, 4, and 6 (*θ*_s_ = 25°). Lines denote the averages of all combinations of leaf angle distribution (LAD) and *S*_l_ types (solid lines), erectophile and *S*_l_ = 0.0001 (dotted lines), and planophile and *S*_l_ = 0.1 (dashed lines). *R*^2^_e_ represents *R*^2^ when the LAD is erectophile and *S*_l_ = 0.0001, and *R*^2^_p_ represents *R*^2^ when LAD is planophile and *S*_l_ = 0.1.

However, for each of the estimated LAI ranges, as the LAD became more vertical or as *S*_l_ decreased, *C*_ab_, *C*_m_, and *C*_w_ decreased for the same canopy spectral reflectance values (Fig. 3). As the relationships between leaf constituents and associated canopy reflectances were dependent on the LAD and *S*_l_ types, it was difficult to estimate *C*_ab_ and *C*_w_ from the canopy spectral reflectances when different LAD and *S*_l_ types existed in the focal region of analysis. *R*_p_^2^ and *R*_e_^2^ in Figure 3 were the coefficients of determination under erectophile and *S*_l_ = 0.0001 parameterization and under planophile and *S*_l_ = 0.1 parameterization, respectively. In contrast, for the estimation of the *C*_w_/*C*_ab_ ratio, estimation equations were independent of the LAD and *S*_l_ types (Fig. 3). The relationships between the estimated and observed *C*_w_/*C*_ab_ ratios for all combinations of the LAD and *S*_l_ types (Table 1) fell within the two regression curves for erectophile and *S*_l_ = 0.0001 parameterization and for planophile and *S*_l_ = 0.1 parameterization (Fig. 3).

I found that the models were not sensitive to the value used for *θ*_s_. For an LAI value of 1, the *R*^2^ of *C*_ab_, *C*_w_, and *C*_w_/*C*_ab_ estimates were <0.30, and the relationship between *C*_m_ and the canopy spectral reflectance was weak. I could not estimate *C*_ab_ and *C*_w_ when different LAD and *S*_l_ types existed in the focal region of analysis because the equations used to estimate *C*_ab_ and *C*_w_ were dependent on the LAD and *S*_l_ types. However, the estimation equations for *C*_w_/*C*_ab_ were independent of the LAD and *S*_l_ types. Coefficients of the regression equations of *C*_w_/*C*_ab_ for all combinations of LAD and *S*_l_ types together in the form of equation (3) and the *R*^2^ are shown in Table 2.

**Table 2:** Coefficients of the equations for estimating *C*_w_/*C*_ab_

*θ*_s_	*LAI*	*a*_1_	*b*_1_	*b*_2_	*a*_2_	*c*_1_	*c*_2_	*R*^2^
25	2	12.231	1.003	1.659	0.177	0.859	0.691	0.58
	3	8.660	0.949	1.541	0.349	0.858	0.847	0.67
	4	6.632	0.903	1.446	0.443	0.781	0.845	0.72
	5	5.392	0.866	1.373	0.665	0.682	0.876	0.77
	6	4.604	0.841	1.326	1.300	0.587	0.981	0.83
	7	4.179	0.784	1.286	3.604	0.529	1.208	0.89
50	2	12.120	0.978	1.645	0.239	0.898	0.785	0.61
	3	8.776	0.914	1.525	0.400	0.881	0.888	0.67
	4	6.745	0.870	1.430	0.532	0.807	0.900	0.73
	5	5.727	0.831	1.369	0.937	0.706	0.971	0.78
	6	4.932	0.810	1.327	2.136	0.614	1.117	0.84
	7	4.549	0.726	1.281	7.318	0.535	1.391	0.90

## Discussions and Conclusion

I evaluated global estimates of *C*_ab_, *C*_m_, *C*_w_, and *C*_w_/*C*_ab_ from TOA broadband spectral reflectance using the PROSAIL model for possible distributions of leaf and soil spectra, LAI, canopy geometric structure, and leaf size. For LAI values <2, *C*_ab_, *C*_w_, and *C*_w_/*C*_ab_ had weak relationships with their associated spectral reflectances. For LAI values greater than 2, *C*_ab_ and *C*_w_ could be reliably estimated only for certain ranges of LAD and *S*_l_ types while the ratio of *C*_w_/*C*_ab_ could be reliably estimated for all possible canopy structures with an *R*^2^ ranging from 0.56 to 0.90. The estimation equation of *C*_w_/*C*_ab_ from the spectral reflectance and the *R*^2^ value were dependent on the LAI values, but independent of the LAD and *S*_l_ types.

Levels of *C*_ab_, *C*_m_, and *C*_w_ can be indicative of leaf physiology and plant condition, and attempts have been made to estimate these values with remote sensing applications (Ustin et al. 2009). In previous site-specific studies, *C*_ab_, *C*_m_, and *C*_w_ were successfully estimated using this methodology (Fourty and Baret 1997; Bowyer and Danson 2004; Zarco-Tejada et al. 2004, 2003; Gitelson et al. 2005; De Santis et al. 2006; Darvishzadeh et al. 2012; Si et al. 2012); however, the use of remote sensing to estimate these values at regional and global scales has not been reported. My research suggests that estimates of *C*_ab_, *C*_m_, and *C*_w_ are dependent on LAD and *S*_l_ types, and these types often vary across regional to global scales. As techniques for estimating LAD and *S*_l_ types through remote sensing have not been established, a generalized estimation of *C*_ab_, *C*_m_, and *C*_w_ across broad spatial scales is difficult except in cases where specific LAD and *S*_l_ types can be inferred. In contrast, this study shows that the estimation of the *C*_w_/*C*_ab_ ratio through remote sensing techniques is generally possible across regional and global scales. The estimation of this ratio through remote sensing has not been previously considered as an indicator of plant canopy condition because the physiological meaning of the *C*_w_/*C*_ab_ ratio has been less well studied than those of *C*_ab_, *C*_m_, and *C*_w_. However, I found that the *C*_w_/*C*_ab_ ratio had a stronger relationship with its associated spectral band than *C*_ab_, *C*_m_, and *C*_w_ had to their associated bands.

Previous studies described the meaning behind and variations in *C*_ab_, *C*_w_, and *C*_w_/*C*_ab_. The value of *C*_w_/*C*_ab_ is specific to plant species, although, in general, the ratio slightly decreases with spring sprout and increases with autumn defoliation (Gond et al. 1999; Ceccato et al. 2001). For example, Scots pine, lodgepole pine, sun flowers, and sugar beets have high *C*_w_/*C*_ab_ values (1.0–1.8); poplars, oaks, and rhododendrons have moderate ratio values (0.5–0.7); and maize and rice have low values (0.1–0.2; Ceccato et al. 2001; Gond et al. 1999; Hosgood et al. 1995). The value of *C*_w_/*C*_ab_ also generally increases when a plant responds to stressors related to water deprivation (Zhang and Kirkham 1996; Guerfel et al. 2009), heat (Jeon et al. 2006), chilling (Bacci et al. 1996; Jeon et al. 2006; Korkmaz et al. 2010), high light conditions (Jagtap et al. 1998), ultraviolet rays (Alexieva et al. 2001), high salinity (Jaleel et al. 2008; Dogan 2011), and heavy-metal contaminants (Anuradha and Rao 2009). The increase in the ratio as a result of plant stress is caused by a greater decrease in leaf chlorophyll compared with leaf water.

Therefore, a change in the *C*_w_/*C*_ab_ ratio through time is the result of either changes in species composition or changes in the response of plants to stress. The former generally occurs at a yearly to decadal scale, whereas the latter occurs at a daily to monthly scale. Although disturbances such as wildfires, insect attacks, and deforestation can cause an immediate change in species composition, an analysis of changes in LAI and NDVI can help to distinguish between the causes of changes in *C*_w_/*C*_ab_. Thus, the remote estimation of the *C*_w_/*C*_ab_ ratio from satellites offers information on plant status at a global perspective.

Other than leaf chlorophyll *a* + *b*, leaf anthocyanins absorb the green light and generate the errors in the estimation of the *C*_w_/*C*_ab_ ratio. Leaf anthocyanins appear when the leaves of deciduous trees turned red in autumn or under chilling stress (Bacci et al. 1996). When a change in the estimated *C*_w_/*C*_ab_ ratio possibly appeared in such cases, an analysis of changes in the estimated LAI and NDVI can help to distinguish between the causes of changes in the estimated *C*_w_/*C*_ab_, as the autumn coloration reduces NDVI (Zhang et al. 2012).

This study modeled the global vegetation as to obey PROSAIL model with parameters shown in Table 1. The *R*^2^ of the LAI estimation from NDVI with globally fixed equations were 0.30–0.39, whereas the *R*^2^ of the *C*_w_/*C*_ab_ ratio estimation in the way I presented in this note was 0.56–0.90, for the estimated LAI values >2. This indicates that the global remote estimation of the *C*_w_/*C*_ab_ ratio is more reliable than that of LAI.
